# Bispecific antibody simultaneously targeting PD1 and HER2 inhibits tumor growth via direct tumor cell killing in combination with PD1/PDL1 blockade and HER2 inhibition

**DOI:** 10.1038/s41401-021-00683-8

**Published:** 2021-05-14

**Authors:** Chang-ling Gu, Hai-xia Zhu, Lan Deng, Xiao-qing Meng, Kai Li, Wei Xu, Le Zhao, Yue-qin Liu, Zhen-ping Zhu, Hao-min Huang

**Affiliations:** Sunshine Guojian Pharmaceutical (Shanghai) Co. Ltd., 3SBio Inc. Company, Shanghai, 201203 China

**Keywords:** HER2, Trastuzumab, PD1 blockade, bispecific antibody, antibody-dependent cellular cytotoxicity (ADCC), PD1 immunological synapse

## Abstract

Immune checkpoint blockade has shown significant clinical benefit in multiple cancer indications, but many patients are either refractory or become resistant to the treatment over time. HER2/neu oncogene overexpressed in invasive breast cancer patients associates with more aggressive diseases and poor prognosis. Anti-HER2 mAbs, such as trastuzumab, are currently the standard of care for HER2-overexpressing cancers, but the response rates are below 30% and patients generally suffer relapse within a year. In this study we developed a bispecific antibody (BsAb) simultaneously targeting both PD1 and HER2 in an attempt to combine HER2-targeted therapy with immune checkpoint blockade for treating HER2-positive solid tumors. The BsAb was constructed by fusing scFvs (anti-PD1) with the effector-functional Fc of an IgG (trastuzumab) via a flexible peptide linker. We showed that the BsAb bound to human HER2 and PD1 with high affinities (EC_50_ values were 0.2 and 0.14 nM, respectively), and exhibited potent antitumor activities in vitro and in vivo. Furthermore, we demonstrated that the BsAb exhibited both HER2 and PD1 blockade activities and was effective in killing HER2-positive tumor cells via antibody-dependent cellular cytotoxicity. In addition, the BsAb could crosslink HER2-positive tumor cells with T cells to form PD1 immunological synapses that directed tumor cell killing without the need of antigen presentation. Thus, the BsAb is a new promising approach for treating late-stage metastatic HER2-positive cancers.

## Introduction

The HER2/neu oncogene encodes an epidermal growth factor receptor (EGFR)-related receptor, HER2, an orphan receptor whose ligand has not yet been identified. HER2 dimerizes with other HER2 family members to activate downstream signaling pathways and thus plays important roles in cell growth [1, 2]. It has been shown that HER2 is overexpressed in 20%–25% of invasive breast cancer patients and its overexpression is associated with more aggressive diseases and poor prognosis [3, 4].

Trastuzumab (Herceptin), a recombinant humanized monoclonal antibody (mAb) against HER2 developed by Roche/Genentech, has become the standard of care for patients with HER2-positive breast cancer over decades [5–7]. However, the objective response rates to trastuzumab monotherapy range between 12% and 34% and the majority of patients who initially responded to trastuzumab generally develop resistance or relapse after a year [8]. To this end, alternative anti-HER2 antibody-based therapeutics including antibody-drug conjugates, such as T-DM1 and DS8201s, and bispecific antibodies (BsAbs) are being actively developed and have shown significant clinical benefits in several cancer indications [9–12].

Programmed death-1 (PD1) is an inhibitory receptor expressed in T and B cells as well as myeloid-derived cells [13–16]. Binding of PD1 to its ligand PDL1 (CD274, B7-H1) inhibits T-cell proliferation and cytokine production and, thus, turns them into a state of exhaustion [17, 18]. Anti-PD1 mAbs capable of blocking PD1 and PDL1 have shown promising antitumor activity in a broad spectrum of cancer types in preclinical studies and clinical trials [19–25]. Nevertheless, only a fraction of patents (approximately 20%) achieved durable clinical responses after anti-PD1 antibody monotherapy, while most patents either had no responses at all or exhibited transient responses [26–30].

BsAbs are antibodies comprising two distinct antigen-targeting domains and can be used in place of combination of two mAbs. BsAbs have been extensively studied over years for therapeutic use, because they can provide therapeutic benefits that mAbs cannot [31–35]. A BsAb can bridge its two target antigens and bring them into close proximity [36]. A BsAb can retarget the effector cells, e.g., T cells, against tumor cells by simultaneously binding to cell surface antigens expressed from both cells and thus significantly activate the antitumor activities of effector cells [37–39]. A BsAb can also ligate two different receptors on the same cell and change intracellular downstream signaling [40]. Using antibody engineering technology to create a BsAb targeting HER2 and PD1, we aimed to synergize the antitumor activities rendered by HER2 blockade and PD1/PDL1 blockade.

We report the creation of a BsAb by combining an anti-HER2 antibody IgG and a scFv from an anti-PD1 antibody (609A). The BsAb retained its binding specificity for its original targets and was able to direct T cells to engage HER2-overexpressing tumor cells, resulting in PD1 synapse formation. In addition to the ability to block HER2 signaling and induce antibody-dependent cellular cytotoxicity (ADCC), the BsAb also activated antitumor immunity through PD1/PDL1 blockade in vivo. Importantly, the BsAb exhibited superior tumor cell killing activity in the presence of peripheral blood mononuclear cells (PBMCs) or activated T cells relative to combination of the two parental mAbs. Thus, our anti-HER2×anti-PD1 BsAb represents a new strategy to enhance antibody therapeutic efficacy through tumor-targeted immune checkpoint blockade.

## Materials and methods

### Cell culture

The cell lines used in this study were obtained from American Type Culture Collection (ATCC; Manassas, VA, USA) unless otherwise indicated. The cells were cultured at 37 °C with 5% CO_2_. Cells for protein expression: HEK293E cells were cultured in FreeStyle 293 medium (Life Technologies, Rockville, MD, USA). Cells for bioassays: NCI-N87 or BT474 cells were cultured in RPMI-1640 supplemented with 10% FBS. PD1/CHO-S cells were cultured in CD Forti-CHO supplemented with 1% Glutamax and 0.4% anti-clumping agent. PD-L1 aAPC/CHO-K1 cells (Promega, Cat#J1252, Madison, WI, USA) were cultured in 90% Ham’s F12 (Gibco Cat#11765-054) with 10% FBS, 200 μg/mL hygromycin B (Gibco, Cat#10687-010, Shanghai, China) and 200 μg/mL G418 sulfate solution (Promega, Cat#V8091). PD1 effector cells (Promega, Cat#J1250) were cultured in 90% RPMI-1640, supplemented with 10% FBS, 200 μg/mL hygromycin B, 500 μg/mL G418 sulfate solution, 1% sodium pyruvate (Promega, Cat#11360070), and 1% MEM nonessential amino acids (Gibco, Cat#11140050). Cells for ADCC: NK92-CD16 cells (ATCC, Cat#pta­8837) were cultured in α­MEM medium without nucleosides and with 2 mM glutamine, 0.1 mM 2­-mercaptoethanol, 0.2 mM inositol, 0.02 mM folic acid, 12.5% FBS, 12.5% Horse Serum (ATCC, Cat#302040), and 100 units/mL human recombinant IL­-2. Cells for animal study: MC38 cells (Jennio Biotech, a mouse colon cancer cell line, Guangzhou, China) were cultured in DMEM with 10% FBS; JIMT-1 (Cobioer, Cat#CBP60378, Nanjing, China), a breast cancer cell line, were cultured in DMEM medium supplemented with 10% FBS. PBMCs (Cat#SLB-HP050A) and CD3^+^ beads-isolated T cells (Cat#SLB-CD3T-10AN) were purchased from Sailybio (Shanghai, China) and used when applicable.

### Protein expression and purification

Constructs expressing the anti-HER2×PD1 BsAb variants were generated using the pTT5 vector (NRC Biotechnology Research Institute, Montreal, QC, Canada). The expression vectors were transiently transfected into HEK293E cells using 1 μg/mL 25 kDa linear PEI (Polysciences, Inc.). One day after transfection, valproic acid (Sigma) was added to the cell culture at a final concentration of 3 mM. On day 2 after transfection, medium comprising 10% GlutaMAX, 10% 400 g/L glucose, and 80% freestyle 293 medium was added to the cell culture to 10% of the total volume. Conditioned medium was collected 5–6 days after transient transfection.

BsAbs in the culture medium were purified by MabSelect SuRe (GE) affinity columns using an Akta Avant 25 fast protein liquid chromatography purification system. After equilibrating the column with buffer A (25 mM sodium phosphate, 150 mM sodium chloride, pH 7.4), the culture media containing BsAbs was loaded into the column, which was then eluted with buffer B (100 mM sodium citrate, pH 3.5) to collect the desired proteins. The eluted proteins were neutralized with 1 M Tris-HCl at pH 9.0.

### Flow cytometric analysis of BT474 cells and PD1/CHO cells

To measure the binding affinity of the anti-HER2×PD1 BsAb for HER2-overexpressing cells, BT474 cells (1 × 10^5^/well in 96-well plate) were incubated with three-fold serial dilutions of the BsAb ranging from 3.3 pM to 200 nM in 200 μL PBS at 4 °C for 1 h. Cells were washed three times with PBS and then incubated with FITC-conjugated goat anti-human IgG (Jackson, Cat.#109-095-003) at 4 °C for 1 h. The cells were washed and resuspended in 200 μL PBS and were analyzed on FACS (BECKMAN, Cytoflex).

To measure the binding affinity for PD1-overexpressing cells, PD1/CHO cells were used instead.

### Bridging ELISA

PD1-ECD proteins (in-house, 200 ng/mL) were coated in 96-well plates (Thermo Fisher, Cat#439454) at 4 °C overnight. The plates were washed with PBST (PBS containing 0.05% Tween-20), blocked for 1 h with PBS containing 2% BSA and incubated with three-fold serial dilutions of antibodies for another hour at 37 °C. The plates were then washed three times and incubated with 2 µg/mL His-tagged HER2-ECD for 1 h at 37 °C. After washing, an HRP-conjugated anti-6×HisTag mAb (Invitrogen, Cat#MA1-21315-HRP) was added and incubated for 1 h at 37 °C. The plates were washed and the reaction was developed with TMB substrates. The plates were then read on a SpectraMax 190 reader (Molecular Devices) at 450 nm.

### Proliferation inhibition assay

BT474 cells, a HER2-overexpressing human breast cancer cell line, were seeded at 5000 cells/well in a 96-well culture plate (Costar, Cat#3599) supplemented with RPMI-1640+10% FBS, and incubated overnight at 37 °C with 5% CO_2_. The next day, the cells were incubated with serially diluted anti-HER2×PD1 BsAb or trastuzumab ranging from 8 pM to 150 nM in a final volume of 200 μL/well. The plates were incubated at 37 °C for 6–7 days and viability was quantitated with Cell counting Kit-8 (Dojindo, Cat#CK04). The plates were then read on a SpectraMax 190 reader (Molecular Devices) at 450 nm.

### PD1/PDL1 blockade bioassay

The assay was carried out following the manufacturer’s instruction (Promega, Cat#J1250). Briefly, PD-L1 aAPC/CHO-K1 cells were seeded at 4 × 10^4^ cells/well at 100 μL in white 96-well plates followed by cultured overnight in a 37 °C incubator with 5% CO_2_. The next day, the supernatant was discarded and the PD-L1 aAPC/CHO-K1 cells were incubated with three-fold serial dilutions of the BsAb ranging from 0.04 to 300 nM and PD-1 effector cells (5 × 10^4^/well) in 80 μL assay buffer (99% RPMI-1640 supplemented with *L*-glutamine + 1% FBS) for 6 h. Bio-Glo™ Reagent (80 μL) was added to each well and the bottom was sealed with opaque film. The plate was incubated at ambient temperature for 5–30 min and then luminescence was measured using a SpectraMax i3x. The expression of luciferases under the control of NFAT transcriptional response elements was measured as a readout in response to PD1/PDL1 blockade.

### ADCC assay

BT474 cells were seeded in 96-well flat-bottom plates at a density of 1 × 10^4^ cells/well at 50 μL in RPMI-1640 supplemented with 5% FBS. NK92a cells (4 × 10^4^/well, Cat#pta­8837) were added to each well in the presence of serially diluted anti-HER2×PD1 BsAbs and control antibodies for a final reaction volume of 150 μL, and the plates were incubated for 3 h at 37 °C and 5% CO_2_. Three hours later, 100 μL supernatant was transferred to new plates. To monitor cell lysis, 50 μL LDH substrate was added to each well containing the supernatant and the plates were incubated at room temperature for 15 min. The plates were then read at 490 nm on a SpectraMax 190 reader (Molecular Devices). The % lysis was converted from *OD* values according to the following formula: (*OD*_sample_ − *OD*_*T*_ − *OD*_nk_)/*OD*_lysis_ × 100%. The EC_50_ was calculated using GraphPad Prism 7 software (GraphPad Software). In the case of T cells, pre-activated T cells with anti-CD3 antibody were used instead of tumor cells.

### Tumor cell killing assay

PDL1-overexpressing N87 cells (N87-PDL1) were diluted to 1 × 10^5^ cells/mL in RPMI-1640 supplemented with 1% FBS. N87-PDL1 cells (100 μL/well) were seeded into the wells of 96-well plates and incubated overnight at 37 °C and 5% CO_2_. The next day, 2, 10, and 50 nM of antibodies [the BsAb, trastuzumab, an anti-PD1 mAb (609A) and trastuzumab+609A] and controls were added to each well containing 5000 PBMCs for a reaction volume of 200 μL/well, and the plates were incubated at 37 °C and 5% CO_2_ for 4 days. Cell-Titer Glo reagent (50 μL/well) was added to the plates followed by incubation at room temperature for 5–10 min. The plates were then read on an MD SpectraMax i3 for luminescence at 500 ms.

In the case of activated T-cell killing assays, NCI-N87 cells were seeded in 96-well plates at 1 × 10^4^ cells/well. The next day, 2 and 9 nM of antibodies and controls were added to each well containing 5000 activated T cells for a reaction volume of 200 μL/well, and the plates were incubated at 37 °C and 5% CO_2_ for 7 days. The plates were then read on an MD SpectraMax i3 for luminescence.

### Immunofluorescence assay

PD1-overexpressing Jurkat T cells were stimulated to enhance PD1 expression. The activated T cells were labeled with 200 nM Alexa Fluor 488 (488)-conjugated anti-HER2×PD1 BsAb (T1) or 488-conjugated anti-PD1 mAb (609A) (T2) for 1 h at room temperature respectively. N87 tumor cells were labeled with 100 nM Alexa Fluor 546 (546)-conjugated anti-HER2 mAb (19H6-Hu) in the presence (A1) or absence (A2) of trastuzumab for 1 h at room temperature, respectively. The 488-labeled T cells were then co-cultured with 546-labeled N87 cells with the following formats T1+A2 or T2+A1 in PBS for 30 min at room temperature followed by transfer into 6-well plates pre-coated with poly-lysine prior to fixation with 4% paraformaldehyde. Images were visualized with an LCAch N 40×/0.55 PhP objective (IX53, Olympus).

### Xenografted and syngeneic tumor models

Animal care and in vivo experiments were approved by the institutional IACUC of Sunshine Guojian Pharmaceutical (Shanghai) Co. Ltd. and performed under approved protocols (approval code for NCI-N87 xenograft model: AS-2018-058; for MC38 syngeneic model: AS-2019-006). NCI-N87 xenograft tumor models were established with 6-week-old female Balb/c nude mice (purchased from Charles River Laboratories) by subcutaneous injection of 8 × 10^6^ NCI-N87 cells mixed with 50% Matrigel. MC38 syngeneic tumor models were established in 18–20 g female PD-1 humanized mice (B6-Pdcd1^em1(hPdcd1)Vst^/Vst, Vital star Biotech) by subcutaneous injection of 1 × 10^6^ MC38 cells. When tumors reached a volume of approximately 150 mm^3^, the animals were randomly divided into groups and i.p. injected twice a week. Tumor volume was measured twice per week and calculated using the formula *V* = *LW*^2^/2 (where *V* = volume, *L* = length, and *W* = width).

In the case of the JIMT-1 xenograft model, JIMT-1 tumors were established in M-NSG mice (NOD.Cg-Prkdc^scid^Il2rg^em1^/Smoc) (Model Organism, Cat# NM-NSG-001) by subcutaneous injection of 8 × 10^6^ JIMT-1 cells mixed with 50% Matrigel. When tumors reached a volume of approximately 200–500 mm^3^, 3 × 10^6^ PBMCs in suspension were intraperitoneally inoculated, and then the animals were randomly divided into groups and treated as described above.

### Statistical analysis

All numerical data, except for mouse xenograft data, are presented as the mean ± standard deviation. Mouse xenograft data are presented as the mean ± standard error. Statistical analysis was performed with GraphPad Prism 7 software and Excel. *P* values were calculated using a two-way ANOVA multiple comparison test. In all tests, differences with *P* values < 0.05 (*) were considered to be statistically significant.

## Results

### Construction and production of the anti-HER2×anti-PD1 BsAb

**Fig. 1:**
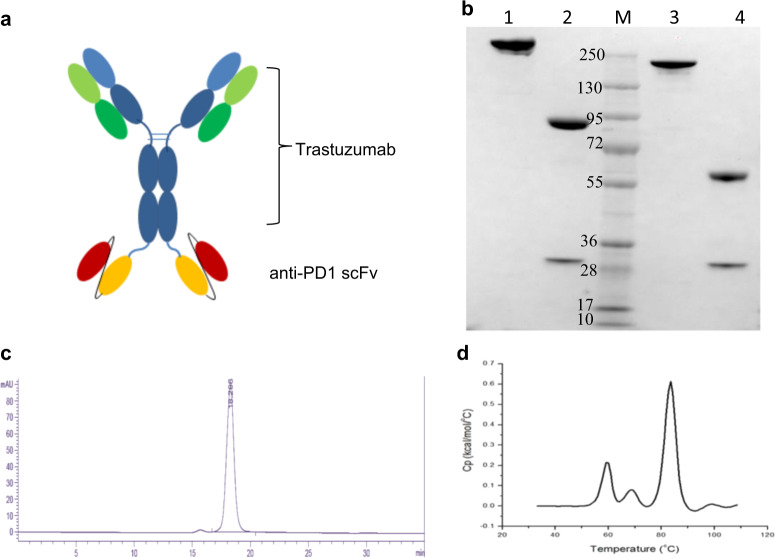
The structure and properties of the anti-HER2×PD1 BsAb. **a** Schematics of the anti-HER2×PD1 BsAb structure. **b** SDS-PAGE showing nonreduced and reduced anti-HER2×PD1 BsAb. Lane 1: nonreduced BsAb; Lane 2: reduced BsAb; Lane 3: nonreduced trastuzumab; Lane 4: reduced trastuzumab; M: Molecular weight markers. **c** SEC chromatogram showing that the BsAb purified by a single-step protein A affinity column had over 95% monomeric species. **d** Differential scanning calorimetry (DSC) of the anti-HER2×PD1 BsAb showing that the antibody has a *T*_onset_ (the temperature at onset of melting) of 52.5 °C and *T*_m_1/2/3 (melting temperatures) of 59.2 °C/68.4 °C/ 83.5 °C, respectively.

The anti-HER2 antibody, trastuzumab, and the anti-PD1 antibody, SSGJ-609A (609A), were utilized as the building blocks to construct the anti-HER2×anti-PD1 BsAb via the IgG-scFv or scFv-IgG fusion format [41–43]. In this format, the scFv of one antibody was fused via a flexible peptide linker [(GGGGS)*n*, *n* = 0–5] to the N- or C-terminus of the heavy chain of the other antibody. Various constructs were examined for their expression levels in transient mammalian culture and their bioactivities in terms of binding to both HER2 and PD1. When the scFv of trastuzumab was fused to either the N- or C-terminus of the heavy chain of the IgG scaffold of 609A, it showed a significantly reduced binding affinity for BT474 cells (a HER2-overexpressing breast cancer cell line), and was much less potent in inhibiting proliferation of the tumor cells, compared to trastuzumab (data not shown). We next used trastuzumab as the IgG scaffold and fused it with the scFv of 609A. Between the two IgG/scFv fusion orientations examined, the BsAb constructed with the 609A scFv fused to the N-terminus of trastuzumab showed ~5-fold lower binding affinity for BT474 cells than did the BsAb with the 609A scFv fused to the C-terminus (Supplementary Fig. S1). Thus, the BsAb with the two copies of 609A scFvs fused to the C-terminus of trastuzumab, namely anti-HER2×PD1 BsAb, was selected for further characterization (Fig. 1a). As demonstrated by SDS-PAGE, SEC-HPLC, and differential scanning calorimetry, the anti-HER2×PD1 BsAb exhibited favorable chemophysical properties as a drug candidate (Fig. 1b–d).

### The anti-HER2×PD1 BsAb simultaneously bound to HER2 and PD1 comparable to the parent monoclonal antibodies

**Fig. 2:**
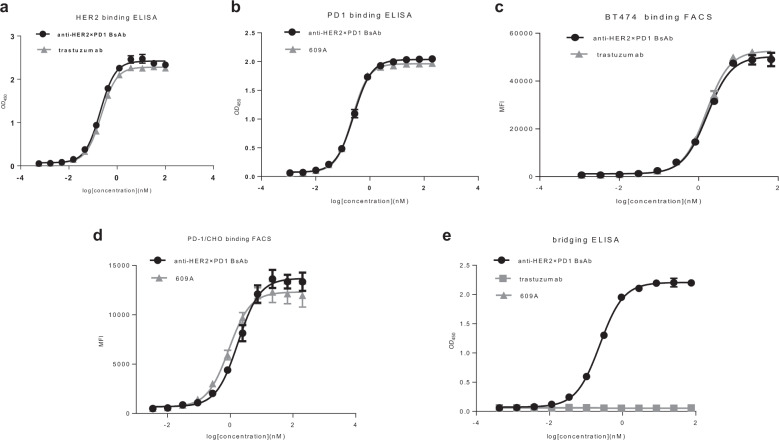
The anti-HER2×PD1 BsAb simultaneously bound to PD-1 and HER2. **a** The binding affinity of the BsAb for HER2 was measured by ELISA. Trastuzumab was used as the positive control. **b** The binding affinity of the BsAb for PD1 was measured by ELISA and compared to that of the parental anti-PD1 mAb, 609A. **c** The ability of the BsAb to bind to BT474, a HER2-overexpressing cancer cell line, was measured by FACS and compared to that of trastuzumab. **d** The ability of the BsAb to bind to PD1-overexpressing CHO cells was measured by FACS and compared to that of the parental anti-PD1 mAb, 609A. **e** A bridging ELISA was set up such that PD1 proteins were coated on the plates followed by the sequential addition of the indicated antibodies and His-tagged HER2 proteins. Anti-6×HisTag monoclonal antibody-HRP was added to visualize the positive binders. The results confirm that the BsAb is capable of simultaneously crosslinking its two targets, HER2 and PD1.

The anti-HER2×PD1 BsAb dose-dependently bound to HER2 and PD1 as shown by ELISA. The EC_50_ (the antibody concentration required for 50% of maximum binding) of the BsAb for HER2 was 0.2 nM. This was comparable to that of trastuzumab, which was 0.22 nM. Similarly, the EC_50_ of the BsAb for human PD1 was 0.14 nM, which was comparable to the EC_50_ of the parental anti-PD1 mAb, 609A (0.15 nM, Fig. 2a, b). The BsAb also bound efficiently to the receptors on the cell surface as shown by FACS analysis. The EC_50_ of the BsAb binding to BT474 cells was 1.64 nM, comparable to trastuzumab, which bound to the same cells with an EC_50_ of 1.56 nM. The EC_50_ of the BsAb binding to PD1-overexpressing CHO cells was 1.78 nM, which was comparable to the EC_50_ of the anti-PD1 mAb, 609A (1.62 nM, Fig. 2c, d). In addition to binding to PD1-overexpressing cell lines, we also tested the ability of the BsAb to bind to primary T cells. As expected, the BsAb was indeed capable of binding to activated primary T cells (Supplementary Fig. S2). To confirm that the BsAb can simultaneously bind to its two targets, a bridging ELISA was performed and the results showed that the BsAb was capable of crosslinking HER2 and PD1 with an EC_50_ of 0.24 nM, whereas the crosslinking activity was not achieved with the parental mAbs, trastuzumab and 609A (Fig. 2e).

### The anti-HER2×PD1 BsAb retained the full biological activities of the respective parental antibodies in cell-based assays

**Fig. 3:**
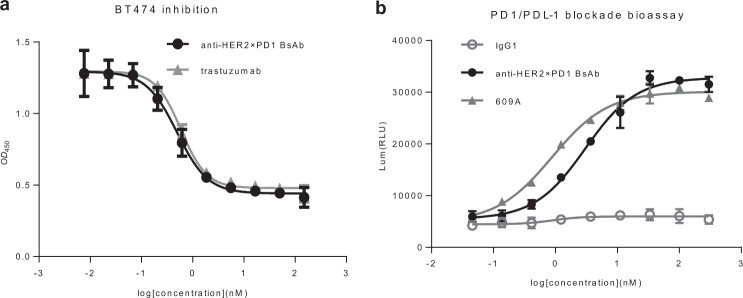
The anti-HER2×PD1 BsAb inhibited the proliferation of HER2-overexpressing tumor cells and blocked the PD1/PDL1 interaction in cell-based bioassays. **a** The BsAb inhibited the proliferation of HER2-overexpressing BT474 cancer cells in a dose-dependent manner similar to trastuzumab. **b** The ability of the BsAb to block PD1/PDL1 signaling was measured and compared to that of the parental anti-PD1 mAb, 609A, using a PD1/PDL1 blockade cell-based assay, in which the expression of luciferases were monitored under the control of nuclear factor of activated T cells (NFAT) response elements in response to blockade of PD1/PDL1 signaling (Promega Cat#J1250). A nonspecific IgG1 was used as the negative control.

In tumor cell growth assays, the BsAb effectively inhibited the proliferation of HER2-overexpressing BT474 cells at an IC_50_ (the antibody concentration inhibiting 50% of cell proliferation) of 0.59 nM on par with that of trastuzumab whose IC_50_ was 0.5 nM (Fig. 3a). Similarly, the BsAb potently blocked PD1/PDL1 cell signaling with an IC_50_ of 3.28 nM, which was comparable to that of 609A (0.89 nM, Fig. 3b). Thus, the BsAb retained its ability to inhibit HER2-overexpressing tumor cell growth via HER2 blockade and to activate T cells via PD1 blockade.

### The anti-HER2×PD1 BsAb exhibited ADCC toward HER2-overexpressing tumor cells but not T cells

**Fig. 4:**
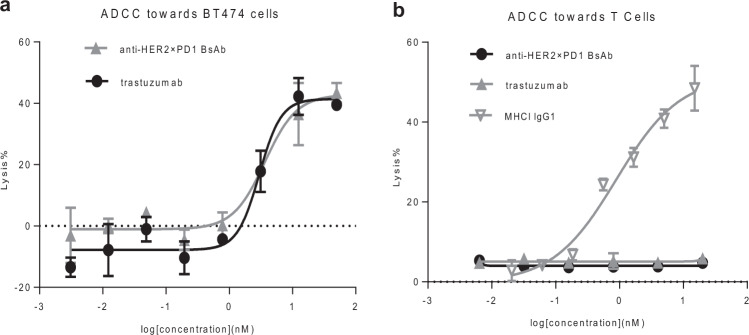
The anti-HER2×PD1 BsAb retained ADCC toward tumor cells but not T cells. **a** The BsAb exhibited a potency in lysing tumor cells similar to that of trastuzumab in the ADCC assay. **b** The BsAb and trastuzumab failed to mediate ADCC toward T cells, whereas an anti-MHC1 IgG1 antibody showed strong potency in lysing T cells in a dose-dependent manner [44].

ADCC plays an important role in trastuzumab-mediated tumor cell killing. Thus, we tested the BsAb for ADCC toward cancer cells and T cells, as we would like to confirm whether the scFvs (anti-PD1) fused to the effector-functional Fc of an IgG (trastuzumab) by linkers still possessed ADCC activity. The BsAb exhibited strong ADCC toward BT474 tumor cells, comparable to that of trastuzumab (Fig. 4a). By contrast, no ADCC toward T cells could be detected, while a control antibody targeting major histocompatibility complex (MHC) I of T cells exhibited potent ADCC toward T cells [44] (Fig. 4b). This result is in line with the previous finding where a BsAb with anti-PD1 scFvs fused to the N-terminus of cetuximab showed no ADCC toward T cells [39]. This suggests that proper spacing between the cell surface receptors and the Fc region of an IgG molecule or a favorable protein configuration as a whole, such as an intact IgG format, might be a critical determinant for initiating ADCC.

### The anti-HER2×PD1 BsAb exhibits synergistic killing of cancer cells in a PDL1 expression-independent manner ex vivo

**Fig. 5:**
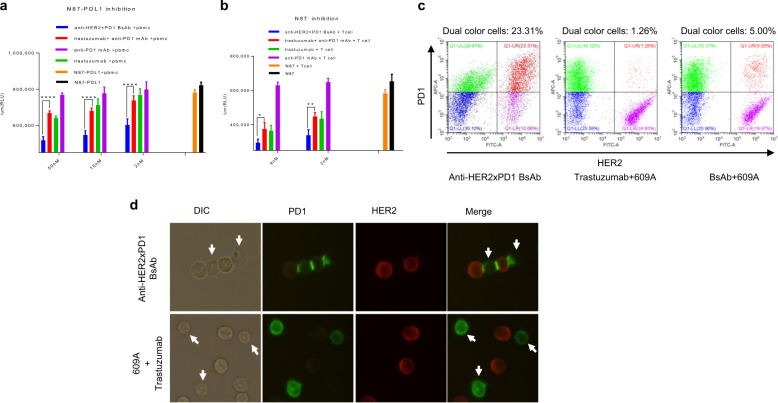
The anti-HER2×PD1 BsAb exhibited synergistic killing effects on cancer cells in a PDL1 expression-independent manner ex vivo. **a** Human PBMCs were mixed with PDL1-overexpressing N87 tumor cells (N87-PDL1) in the presence of 2, 10, and 50 nM indicated antibodies (the N87-PDL1/PBMC pair). **b** Activated Jurkat T cells were mixed with parental N87 tumor cells in the presence of 2 and 9 nM indicated antibodies (the N87/T cell pair). With respect to the combination treatment comprising trastuzumab and 609A, equal molar amounts of the two mAbs (2, 10, and 50 nM each) were combined and added to the cells. The number of viable cells was measured as relative luminescence units (RLUs). The different treatment groups were as follows: N87-PDL1 (N87), N87-PDL1 (N87) tumor cells only; N87-PDL1+PBMCs (N87+T cells), N87-PDL1 cells mixed with PBMCs (N87 cells mixed with T cells) in the absence of antibodies; Anti-PD1 mAb+PBMCs (Anti-PD1 mAb+T cells), N87-PDL1 cells mixed with PBMCs (N87 cells mixed with T cells), and the anti-PD1 mAb, 609A; Trastuzumab+PBMCs (Trastuzumab+T cells), N87-PDL1 cells mixed with PBMCs (N87 cells mixed with T cells) and trastuzumab; Trastuzumab+anti-PD1 mAb+PBMCs (Trastuzumab+anti-PD1 mAb+T cells), combination of trastuzumab and 609A added to N87-PDL1 cells in the presence of PBMCs (N87 cells in the presence of T cells); and Anti-HER2×PD1 BsAb+PBMCs (Anti-HER2×PD1 BsAb+T cells), the BsAb added to N87-PDL1 in the presence of PBMCs (N87 cells in the presence of T cells). *P < 0.05, **P < 0.01, and ****P < 0.0001 by two-way ANOVA. **c** PD1-overexpressing Jurkat T cells prelabeled with an Alexa Fluor 647-conjugated anti-PD1 mAb (Sinobiological, Cat#MM18) were mixed with N87 cells prelabelled with an Alexa Fluor 546-conjugated anti-HER2 mAb (19H6-Hu) in the presence of the BsAb (left), the mixture of trastuzumab and 609A (center), the BsAb plus 609A (right). The group in red (pseudo-color) (upper right corner) shows cells that have dual emission signals (MFI > 1 × 10^4^ for FITC-A and MFI > 3 × 10^4^ for APC-A), meaning that the two types of cells were associated together. The group in green (pseudo-color) (upper left corner) shows T cells labeled with the Alexa Fluor 647-conjugated anti-PD1 mAb (MFI > 3 × 10^4^ for APC-A). The group in purple (pseudo-color) (lower right corner) shows N87 cells labeled with the Alexa Fluor 546-conjugated anti-HER2 mAb (MFI > 1 × 10^4^ for FITC-A). **d** Immunofluorescence microscopy showing costaining of PD1 (green) on T cells and HER2 (red) on N87 tumor cells in samples treated with the BsAb or a mixture of the anti-PD1 mAb (609A) plus trastuzumab. Note that one tumor cell was ligated with two T cells, while a single T cell was in contact with two tumor cells in the presence of the BsAb. The combination of 609A and trastuzumab failed to induce PD1 synapse formation with T cells. The white arrows denote T cells (DIC channel), for which PD1 staining (green channel) were barely seen owing to the rearrangement of PD1 during synapse formation.

Given that the BsAb can crosslink PD1 on T cells and HER2 on tumor cells, we hypothesized that the BsAb may enhance tumor cell killing by bringing T cells into the proximity of tumor cells and potentially inducing PD1 synapse formation as seen in our previous study [39]. We stably transfected NCI-N87 cells, a HER2-overexpressing gastric cancer cell line that does not express PDL1 when cultured in vitro (data not shown), with a PDL1 construct to obtain a PDL1/HER2 double-overexpressing cancer cell line (N87-PDL1) that could potentially respond to trastuzumab and anti-PD1 mAbs. Addition of various concentrations (2–50 nM) of trastuzumab to N87-PDL1 cells in the presence of human PBMCs dose-dependently reduced the number of viable cancer cells relative to antibody-free controls (Fig. 5a). Combinations of trastuzumab and the anti-PD1 mAb, 609A, showed a killing effect similar to that of trastuzumab alone. Importantly, treating cancer cells with the BsAb resulted in a superior reduction of viable cancer cells compared to that of combination of trastuzumab with 609A (*P* < 0.0001; Fig. 5a).

Interestingly, 609A alone did not enhance PBMC-mediated N87-PDL1 cell killing, even though PDL1 was overexpressed by the tumor cells. To confirm that PDL1 on tumor cells was not responsible for the lack of killing and that the enhanced killing mediated by the BsAb was not simply due to PD1/PDL1 blockade-induced T-cell activation, we examined the killing of parental N87 cells that did not express PDL1 with activated Jurkat T cells in the presence of various antibodies. Consistent with the PBMC results, the BsAb effectively killed more tumor cells than did either trastuzumab alone, or the combination of trastuzumab with 609A. Anti-PD1 mAb alone, again, failed to induce measurable tumor cell killing in the absence of PDL1 on tumor cells (Fig. 5b). This finding suggests that the crosslinking of T cells with cancer cells by the BsAb, in contrast to the effects of the anti-PD1 mAb alone, might be sufficient to allow T cells to recognize and initiate the killing of tumors without the need for presentation of tumor-specific antigens to T cells.

We next demonstrated by FACS that the physical association between HER2-overexpressing tumor cells and T cells was indeed established specifically by the BsAb (Fig. 5c and Supplementary Fig. S3). In addition, immunofluorescence microscopy showed that the BsAb bridged T cells with tumor cells and led to formation of PD1 synapses [45, 46]. It is possible that T cell activity could be significantly further enhanced by PD1 synapses, since multiple PD1 synapses could be simultaneously formed on a single T cell. By contrast, no apparent tumor–T cell associations were observed when the cells were incubated with a mixture of trastuzumab and 609A (Fig. 5d). Taken together, the above findings suggest that the physical ligation of cancer cells with T cells by the BsAb might enhance T cell-mediated antitumor effects in a way that cannot be achieved by simply mixing two mAbs together.

### The anti-HER2×PD1 BsAb exhibits potent antitumor activities in both xenograft and syngeneic animal models

**Fig. 6:**
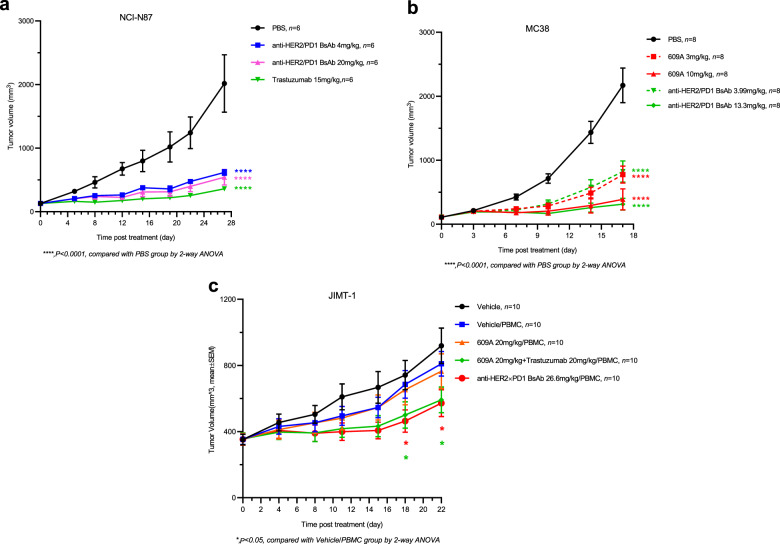
The anti-HER2×PD1 BsAb exhibited potent antitumor effects in vivo. **a** A control (black circle), the BsAb (blue square and pink triangle), and trastuzumab (green triangle) were i.p. injected into mice bearing NCI-N87 tumors at the indicated doses. Tumor volumes (mm^3^) were measured at the indicated time points. **b** A control (black circle), the BsAb (green triangle and diamond), and the anti-PD1 mAb, 609A (red square and triangle) were i.p. injected into human PD1 transgenic mice bearing mouse MC38 tumors at the indicated doses. **c** An isotype control (blue square), 609A (orange triangle), 609A plus trastuzumab (green diamond, 20 mg/kg each), and the BsAb (red circle) were injected into M-NSG mice bearing JIMT-1 tumors in the presence of human PBMCs at the indicated doses. WT mice (black dot) were used as control. Tumor volumes (mm^3^) were measured at the indicated time points. Mean ± SEM. *P < 0.05 by two-way ANOVA (the BsAb/PBMC vs Vehicle/PBMC) and ****P < 0.0001 for the comparison of all indicated groups with the control group.

We next evaluated the antitumor effects of each of the two arms of the BsAb in vivo. First, we measured the efficacy of the BsAb for HER2 blockade in an N87 xenograft mouse model. The BsAb suppressed 75% of N87 tumor growth at a dose of 20 mg/kg on day 28 after treatment. The antitumor efficacy is comparable to that of trastuzumab which inhibited 80% of tumor growth at an equal molar dose of 15 mg/kg (Fig. 6a and Supplementary Fig. S5d).

Second, we analyzed the antitumor activity of the BsAb for PD1 blockade in an MC38 tumor cell line-derived human PD1 transgenic mouse model. The BsAb effectively inhibited ~85% of tumor growth at a dose of 13 mg/kg on day 17 post treatment, on par with that of the anti-PD1 mAb, 609A, where the parent mAb also inhibited 85% of tumor growth at an equal molar dose of 10 mg/kg on the same day (Fig. 6b and Supplementary Fig. S5e). Thus, these results indicated that the anti-HER2×PD1 BsAb retained the antitumor efficacy of each of its arms to a level comparable to that of their parental mAbs in vivo. It is worth noting that no apparent toxicity associated with the BsAb was observed, as the animal body weights kept climbing over the entire course of the treatment (Supplementary Fig. S5a, b).

Since the BsAb does not cross-react with murine PD1 and HER2, we chose a model system in which we introduced human PBMCs into immune-compromised NOD scid gamma mice in order to test the synergistic antitumor activity of the BsAb. Human PBMCs were injected intraperitoneally following the establishment of JIMT-1 tumors in the mice. Although injection of human PBMCs into mice bearing JIMT-1 tumors slightly reduced the body weights initially, the body weights of all groups returned to baseline following treatment (Supplementary Fig. S5c). We then tested the antitumor efficacy of the BsAb in comparison with that of the combination of the two parental mAbs. The anti-PD1 mAb, 609A, failed to show antitumor effects on JIMT-1 tumors relative to the control, whereas 609A in combination with trastuzumab significantly suppressed tumor growth compared to the monotherapy. Importantly, the BsAb also potently inhibited JIMT-1 tumor growth comparable to that of the combination of the two parent mAbs (Fig. 6c and Supplementary Fig. S5f), indicating that the BsAb indeed exhibited dual targeting functions in vivo.

## Discussion

The goal of our study was to combine traditional HER2-targeted therapy with PD1/PDL1 blockade with a novel anti-HER2×PD1 BsAb to treat HER2-overexpressing solid tumors. The BsAb comprises a full trastuzumab IgG with two copies of anti-PD1 (609A) scFvs symmetrically fused to the C-terminus of the heavy chains. The orientation of the IgG and the scFvs was selected based on the finding that the BsAb with scFvs fused to the N-terminus exhibited five-fold reduced binding affinity for HER2 receptors on tumor cells relative to that of the BsAb with scFvs fused to the C-terminus. This is in contrast to an anti-EGFR×PD1 BsAb we previously reported, where fusion of 609A scFvs to the N-terminus of the cetuximab heavy chains was required to keep the binding affinity of the BsAb for target cells intact, in comparison with the parental mAb, cetuximab [39]. Our results emphasize that the geometrical steric hindrance of a receptor on the surface of tumor cells should be carefully assessed during the design of tumor-associated antigen (TAA)-targeting BsAbs.

ADCC plays an important role in trastuzumab-mediated tumor cell killing [47–49]. Ideally, the BsAb is expected to retain full ADCC toward HER2-overexpressing tumor cells while sparing T cells from the attack. Indeed, the BsAb comprising the intact trastuzumab IgG exhibited ADCC toward HER2-overexpressing tumor cells as effectively as parental trastuzumab, whereas the 609A scFv moiety failed to induce measurable ADCC toward T cells, even though the BsAb could effectively bind to PD1 on T cells. Interestingly, we recently reported that the anti-EGFR×PD1 BsAb with 609A scFvs fused to the N-terminus of the cetuximab heavy chains also showed ADCC specific to tumor cells but not to T cells, suggesting that an intact tertiary configuration of an IgG might be a critical determinant for induction of ADCC to kill target cells.

Surprisingly, the anti-PD1 mAb, 609A, in the presence of PBMCs did not enhance the killing of PDL1-overexpressing tumor cells (N87-PDL1) compared to that of PBMCs alone. This was further supported by the finding that the combination of trastuzumab with 609A did not increase the killing of N87-PDL1 cells relative to that of trastuzumab alone in the presence of PBMCs, suggesting that blockade of PD1/PDL1 signaling did not play an important role in T cell-mediated tumor cell killing in this experimental setting. To confirm this hypothesis, we performed tumor cell killing assays with NCI-N87 cells, which do not express PDL1, in the presence of activated T cells. Indeed, the addition of 609A did not enhance killing compared to the addition of T cells alone, nor did it enhance trastuzumab-mediated killing when administered in combination with trastuzumab. In contrast, the BsAb exhibited superior killing of NCI-N87 tumor cells relative to all other treatments. These data imply that BsAb-mediated tumor cell killing may not require presentation of tumor cell antigens to T cells mediated by the MHC as seen in the case of CD3-targeting bispecific T-cell engagers [50]. However, T cells activated by PD1 blockade may still rely on, at least to a certain degree, MHC-dependent antigen presentation for target cell recognition and killing. It is plausible that in addition to the TCR/CD3 complex, crosslinking a TAA with a functional regulatory receptor of T cells, such as PD1, might be sufficient to initiate T cell-dependent target cell killing without the need for MHC-mediated antigen presentation.

In addition to direct tumor cell killing, the BsAb-induced engagement of tumor cells with T cells via HER2 facilitated the formation of PD1 immunological synapses as seen for the anti-EGFR×PD1 BsAb [39]. These results suggest that T-cell activation by PD1 synapse formation and MHC-independent target cell recognition by T cells might be common advantages of a BsAb targeting PD1 and a TAA over the combination of two mAbs for therapeutic use.

Recently, growing evidence has demonstrated that HER2 signaling might be coupled with PD1/PDL1 signaling in HER2-overexpressing tumors [51]. Suh et al. demonstrated that PDL1 expression was more frequent in patients with positive HER2 expression after analysis of over 250 resected tumor tissues. In support of this clinical finding, they also showed that cell lines overexpressing HER2 express a higher level of PDL1 [52]. Interestingly, Fan’s lab showed that targeting HER2 with trastuzumab upregulated the expression of PDL1 via upregulation of IFNγ production, which presumably suppressed antitumor immune responses and led to drug resistance [53]. Thus, a BsAb simultaneously targeting HER2 and PD1/PDL1 signaling pathways might be more effective in treating HER2-overexpressing tumors than the corresponding mAbs. Indeed, our BsAb and a mouse specific BsAb, BsPDL1 × rErbB2, which targets PDL1 and HER2 [54], showed enhanced antitumor activities relative to that of monotherapies with mAbs at equal molar doses in vivo, although the latter is a monovalent BsAb that did not inhibit tumor cell growth in vitro, suggesting that combining HER2 and PD1/PDL1 blockades might improve the treatment of HER2-overexpressing cancers.

In summary, we produced a new anti-HER2×PD1 BsAb. The BsAb was much more cytotoxic to HER2-overexpressing tumor cells in the presence of PBMCs or activated T cells than was trastuzumab either alone or in combination with the parental anti-PD1 antibody, independent of PDL1 expression. We postulate that, in addition to direct HER2 inhibition and ADCC, as is the case with trastuzumab, there are several novel mechanisms that may further contribute to the enhanced cytotoxicity of the BsAb: (1) the BsAb redirects T cells to the proximity of tumor cells and induces direct tumor cell killing most likely in an antigen-presentation-independent manner; (2) the BsAb can potentially activate T cells in the tumor microenvironment via PD1/PDL1 blockade, and (3) the BsAb is capable of further enhancing T cell cytotoxic activity by the formation of PD1 immunological synapses. Thus, we provide a new promising option for treating late-stage or refractory tumors that overexpress HER2.

## Supplementary information

Supplementary Fig. S1

Supplementary Fig. S2

Supplementary Fig. S3

Supplementary Fig. S4

Supplementary Fig. S5

Supplementary Vedio

Supplementary Vedio

Supplementary Vedio
