# Supplementary figures and images for: Bispecific antibody simultaneously targeting PD1 and HER2 inhibits tumor growth via direct tumor cell killing in combination with PD1/PDL1 blockade and HER2 inhibition
Source: Acta Pharmacol Sin. 2021 May 14;43(3):672–80. doi: 10.1038/s41401-021-00683-8 (PMC8888617; doi:10.1038/s41401-021-00683-8)

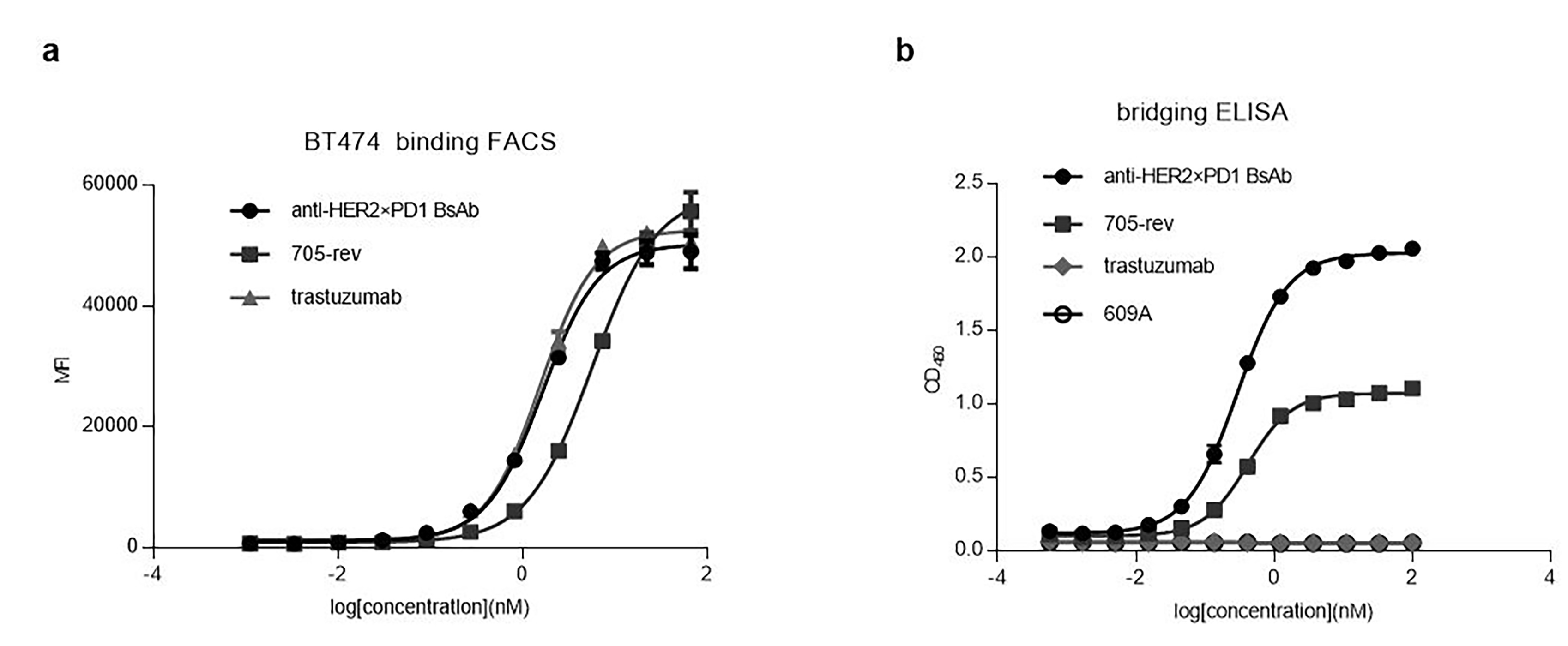

Supplement: Supplementary file 1 — Supplementary Fig. S1 [file 41401_2021_683_MOESM1_ESM.jpg]

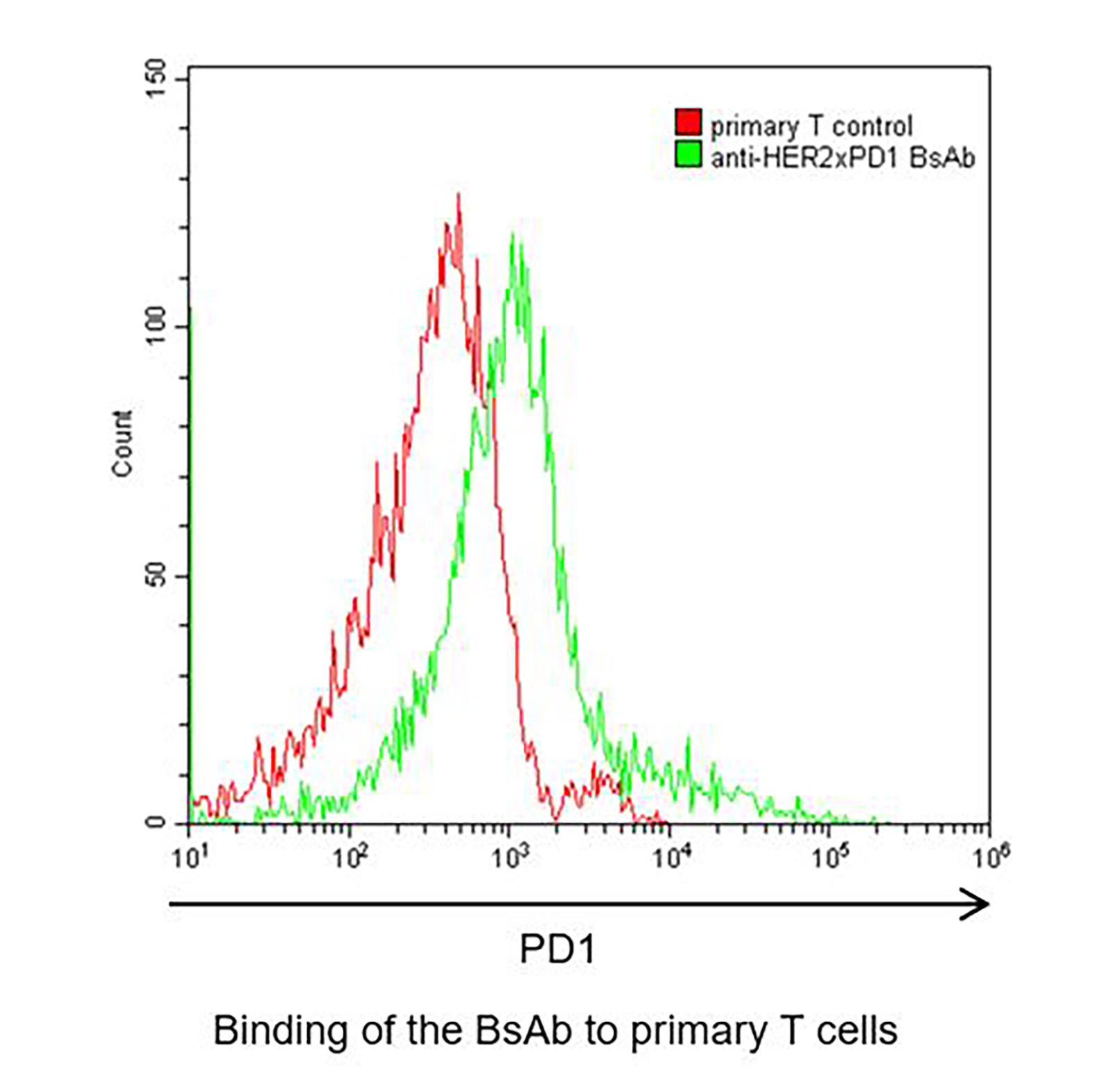

Supplement: Supplementary file 2 — Supplementary Fig. S2 [file 41401_2021_683_MOESM2_ESM.jpg]

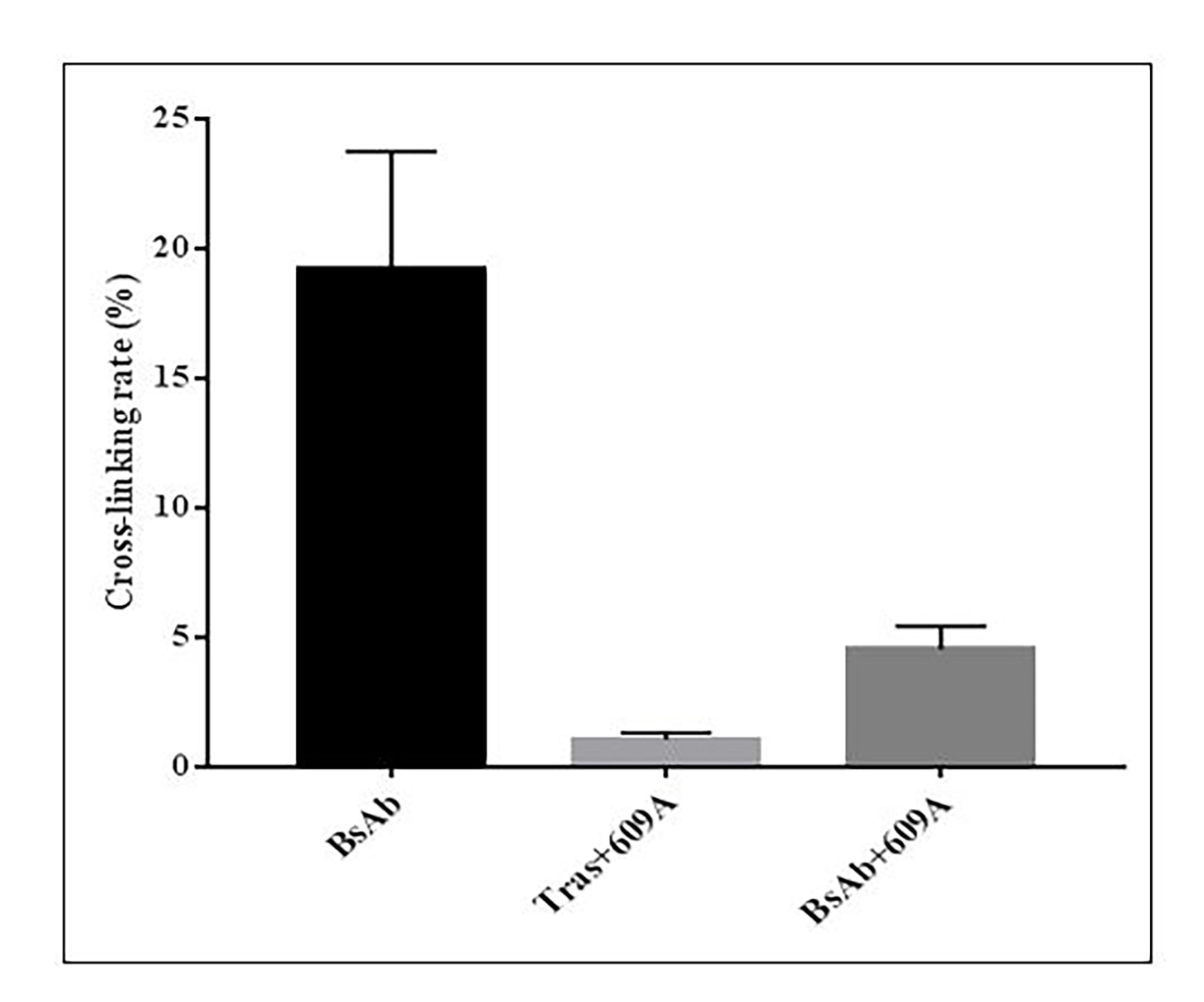

Supplement: Supplementary file 3 — Supplementary Fig. S3 [file 41401_2021_683_MOESM3_ESM.jpg]

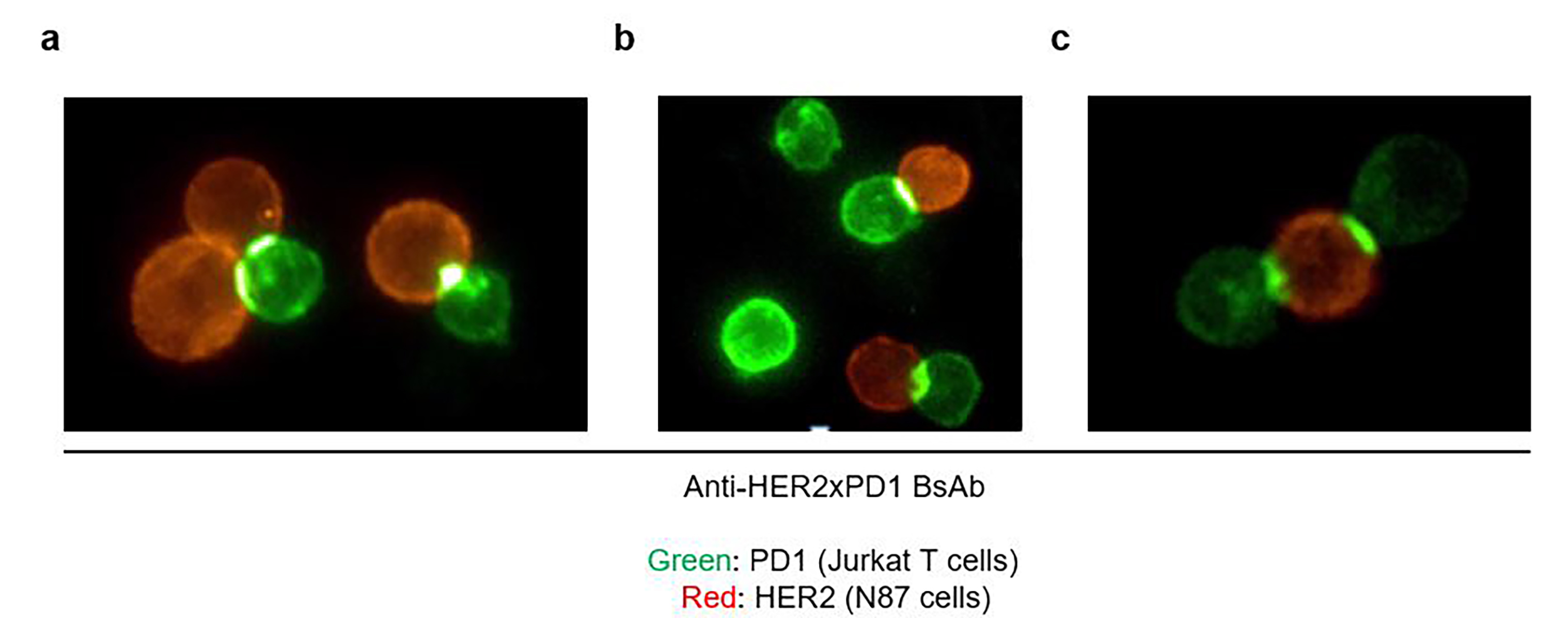

Supplement: Supplementary file 4 — Supplementary Fig. S4 [file 41401_2021_683_MOESM4_ESM.jpg]

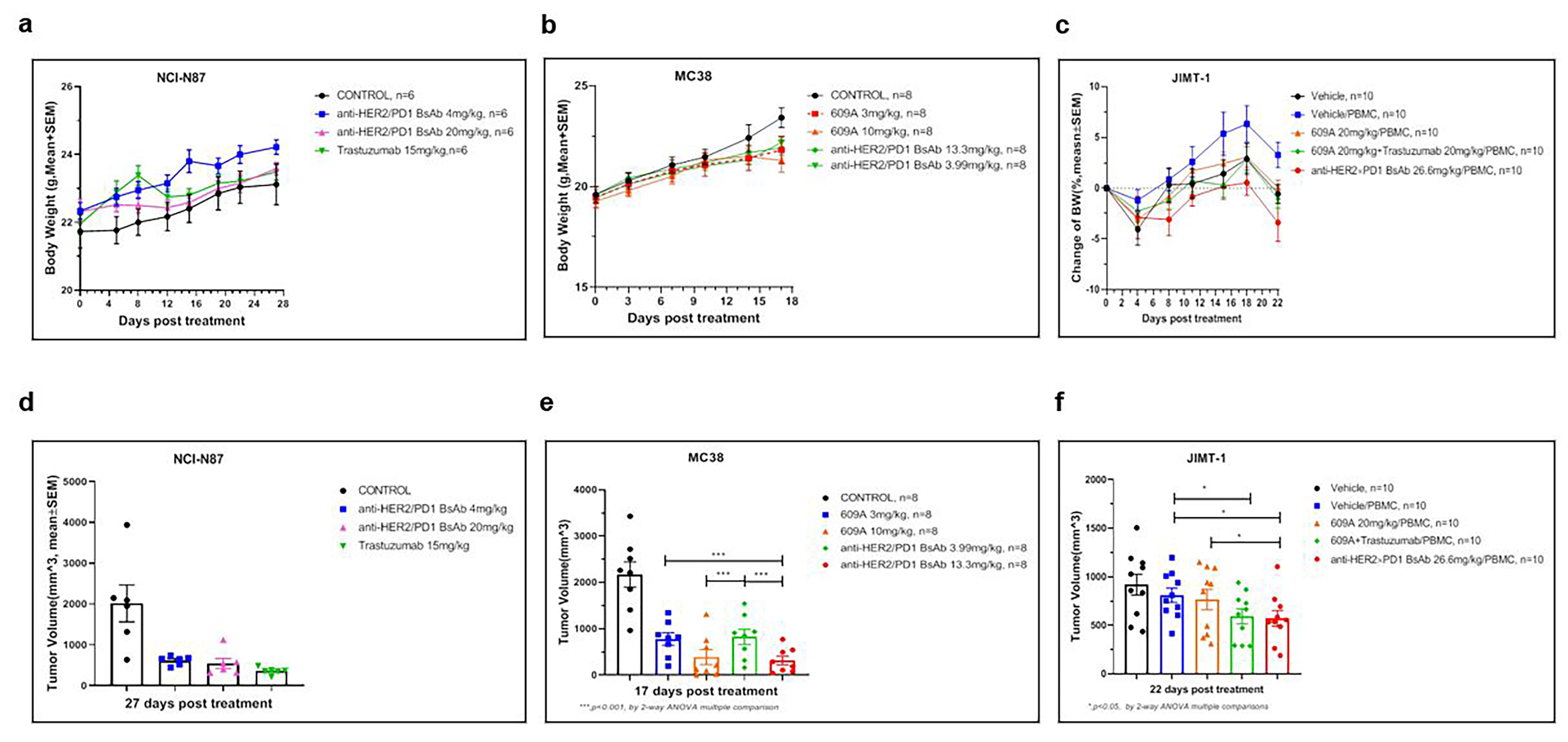

Supplement: Supplementary file 5 — Supplementary Fig. S5 [file 41401_2021_683_MOESM5_ESM.jpg]
